# High Take Off Left Main and Abnormal Origin of Right Coronary Artery: A Case Report

**DOI:** 10.4021/cr256e

**Published:** 2013-05-09

**Authors:** Negar Salehi, Seyfollah Abdi, Hamid Reza Pouraliakba, Anoushiravan Vakili-Zarch

**Affiliations:** a Department of Cardiology, Rajaei Heart center, Tehran University of medical sciences, Iran; b Department of Radiology, Rajaei Heart center, Tehran University of medical sciences, Iran

**Keywords:** Anomalous coronary artery, High take off, Left main, CABG

## Abstract

Coronary anomalies are rare congenital disorders with mostly benign course. We report a case of 54-year-old white male who was with stable angina scheduled for coronary angiography. Due to the difficulty of catheterization, patient underwent CT angiography and high take off left main and right coronary arteries were revealed. We conclude that anomalous coronary arteries are important and coronary interventions may be difficult in their presence.

## Introduction

Anomalies of the coronary arteries are rare congenital disorders. They could be cause of chest pain and in some cases hemodynamic ally significant abnormalities. The development of electrocardiography (ECG) gated multi detector computed tomography (MDCT) helps accurate and noninvasive detection of coronary artery anomalies. Knowing the existence of abnormality can help to choose the correct option for diagnosis and treatment of coronary artery stenosis.

## Case Report

**Figure 1:**
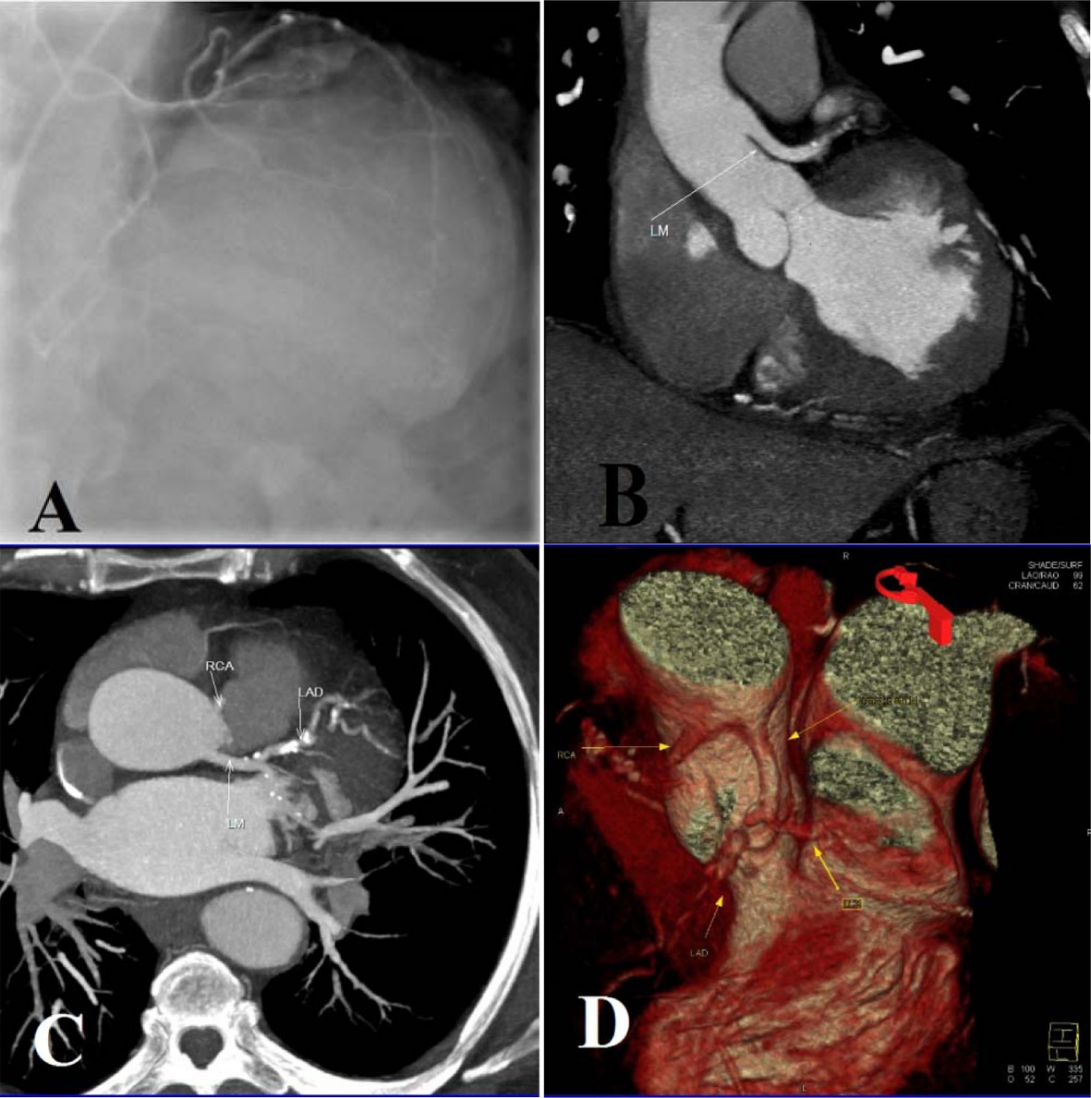
A: shows non selective coronary angiography with separate and same side ostium of left main coronary artery and right coronary artery; B: Origin of left main coronary artery is above sinotubular junction; C and D: proximal left anterior descending artery and mid part of right coronary artery significant stenosis and calcification.

A 54-year-old white male presented to our outpatient clinic with complaint of angina chest pain and dyspnea. He had history of cigarette smoking and family history of coronary artery disease. His physical examination was not remarkable. On electrocardiography there was ST-depression in anterior leads. Myocardial perfusion scan performed and showed moderate to severe ischemia in anterior and inferior territories. Patient admitted and scheduled for coronary angiography. Several attempts to cannulate the coronary arteries were unsuccessful. Aortic root injection showed right coronary artery (RCA) originating from left aortic cusp near left main coronary artery and both are above sinotubular junction (Fig. 1A).On computed tomography coronary angiography left main demonstrated high take off which resulted in long left main artery (Fig. 1B). Left anterior descending artery (LAD) and left circumflex artery (LCX) showed normal course. There was intimal calcification and significant stenosis in proximal of LAD. RCA originated from left cusp and had separate ostium. There was significant stenosis in mid portion of RCA (Fig. 1C, D). Patient underwent coronary artery bypass grafting. He discharged in good condition after 4 days.

## Discussion

Anomalies of the coronary arteries found incidentally in about 1% of healthy individuals [1]. The clinical course of these variations are mostly benign although study on nontraumatic sudden death among young adults revealed that coronary anomalies is the most common cardiac anomaly among them (about 30%) [2].The diagnosis of coronary artery anomalies previously made with angiography. However, recent reports found that correct identification of these anomalies is feasible in only 53% of cases compared to CT angiography study [3]. According to modified classification of Greenberg et al [4] coronary artery anomalies classified into three main groups; anomalous origin, anomalous course and anomalous termination. Anomalous origin group includes high take off arteries, multiple ostia, origin from pulmonary artery and origin from opposite sinus with abnormal course. In our case left main coronary artery and right coronary artery originated from left aortic side and above sinotubular junction correlate with high take off definition. The clinical course of this anomaly assumed to be benign and except for cases with coronary atherosclerosis no intervention is needed. Major clinical problem in these cases is cannulating the artery in attempting for coronary intervention. Due to difficult catheterization CT angiography is recommended [5].

### Conclusion

High take off coronary arteries are regarded as benign conditions but when accompanied by coronary stenosis diagnostic and therapeutic procedures get complicated. Coronary angiography is difficult and coronary interventions are sometimes unsuccessful. Choice of revascularization is bypass grafting and for diagnosis CT angiography is recommended.
